# Successful Multimodality Endoscopic Treatment of Gastric Outlet Obstruction Caused by an Impacted Gallstone (Bouveret's Syndrome)

**DOI:** 10.1155/2008/471512

**Published:** 2007-11-27

**Authors:** Jason N. Rogart, Melissa Perkal, Anil Nagar

**Affiliations:** ^1^Section of Digestive Diseases, Department of Internal Medicine, Yale University School of Medicine, West Haven VAMC, CT 06510, USA; ^2^Department of Surgery, Yale University School of Medicine, West Haven VAMC, CT 06510, USA

## Abstract

Bouveret's syndrome is a rare condition of gastric outlet obstruction resulting from the migration of a gallstone through a choledochoduodenal fistula. Due to the large size of these stones and the difficult location in which they become impacted, endoscopic treatment is unsuccessful and most patients require surgery. We report the case of an elderly male who presented with nausea and hematemesis, and was found on CT scan and endoscopy to have an obstructing gallstone in his duodenal bulb. After several endoscopic sessions and the use of multiple instruments including a Holmium: YAG laser and electrohydraulic lithotripter, fragmentation and endoscopic removal of the stone were successful. We believe this to be the first case of Bouveret's syndrome successfully treated by endoscopy alone in the United States. We describe the difficulties encountered which necessitated varied and innovative therapeutic techniques.

## 1. INTRODUCTION

Gastric outlet obstruction by a gallstone migrating through a cholecystoduodenal fistula (Bouveret's syndrome) is a very rare condition, accounting for fewer than 5% of cases of gallstone ileus [1]. Patients are often elderly with underlying comorbidities, which poses significant challenges to both surgeons and endoscopists. Though there have been several cases of Bouveret's syndrome reported in the literature, there are only a few descriptions of successful treatment with endoscopy alone. We report an unusual case of a large obstructing gallstone trapped in the duodenal bulb and we discuss our successful endoscopic management using multiple therapeutic modalities.

## 2. CASE REPORT

**Figure 1:**
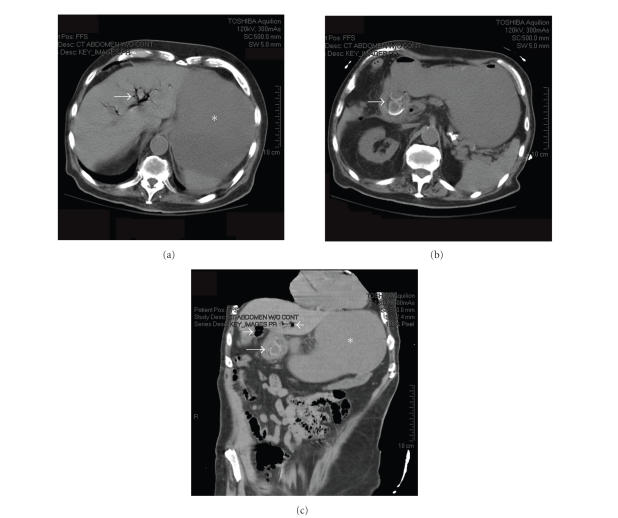
Computed tomography (CT) scan of abdomen and pelvis. (a) Axial image showing pneumobilia (arrow) and a dilated fluid-filled stomach (${}^{*}$). (b) 1-2 large gallstones (arrow) can be seen within an area of inflammation where the gallbladder is in close proximity to the duodenum. (c) Coronal reconstruction showing gallstone within duodenum (long arrow), Pneumobilia (short arrows), and dilated stomach (${}^{*}$) are also seen.

An 85-year-old male with advanced Alzheimer's dementia, diabetes mellitus, and atrial fibrillation presented with several days of nausea, vomiting, and lethargy. There was no report of abdominal pain, fever, or chills. His vital signs were stable and his abdominal exam benign. Nasogastric lavage was significant for one liter of coffee-ground material. His laboratory examination demonstrated a white blood cell count of 25 000/cm^2^, Hematocrit of 33%, creatinine of 1.5 mg/dL, and normal liver enzymes. A CT scan (see Figure 1) showed a markedly distended stomach, air in the biliary tree, and a thickened gallbladder containing one or two large gallstones, the largest 3×2.5 cm in size, which appeared to be abutting the duodenal wall in an area of significant inflammation.

**Figure 2:**
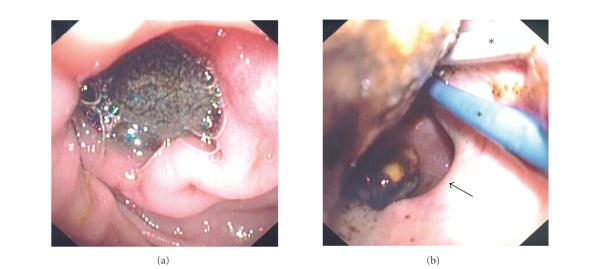
(a) large gallstone in the duodenal bulb, obstructing the pylorus. (b) Attempts to extract the stone failed with multiple instruments, including biliary and CRE balloons (${}^{*}$). The orifice of the choledochoduodenal fistula (arrow) can be seen.

**Figure 3:**
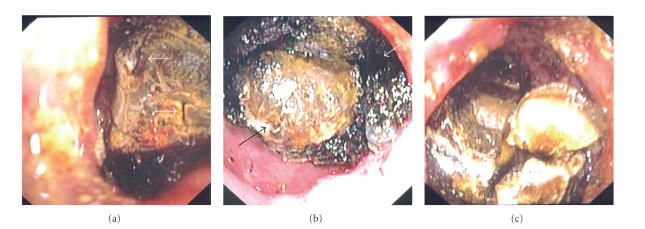
Two different lithotripters were used to fragment the stone. (a) Holmium: YAG laser produced small cracks on the proximal surface (arrows) but the majority of the stone still remained impacted. (b) Electrohydraulic lithotripsy (IEHL) successfully shattered the outer “shell” of the stone (white arrow) and left behind a smaller, much harder core (black arrow). (c) Ultimately, the majority of the stone was fragmented after extensive use of both lithotriptors.

Endoscopy demonstrated old blood in a distended stomach, and a large gallstone in the duodenal bulb obstructing the pylorus (see Figure 2(a)). Due to the patient's advanced age and significant comorbidities, the patient's surgeon advocated endoscopic treatment. The position of the gallstone, the surrounding ulcerated mucosa, and the size of the fistula's orifice made attempts to extraction difficult despite the use of grasping forceps, jumbo biopsy forceps, different-sized and shaped snares, retrieval baskets and nets, as well as biliary balloons and controlled radial expansion (CRE) balloons (see Figure 2(b)). There was no room to maneuver a mechanical lithotripter around the stone, so a Holmium: YAG laser lithotripter was used (Boston Scientific Microvasive, Natick, Mass, USA). A 365-micron laser fiber was passed down one channel of a double-therapeutic gastroscope (GIF 2T160, Olympus America, Inc., Pa, USA), and constant sterile water irrigation was infused through the second channel. Using a total of 3840 joules, 3917 pulses per second for a total time of 7 minutes and 27 seconds, the laser successfully produced small cracks in the stone and ultimately fragmented the proximal portion (see Figure 3(a)); however, the procedure was terminated due to rapid atrial fibrillation and long procedure time.

**Figure 4:**
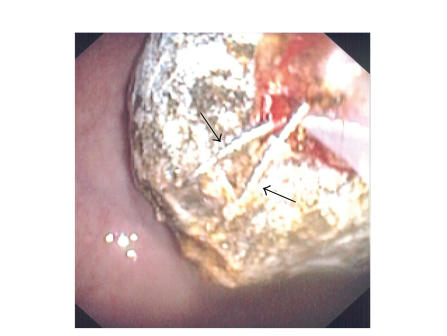
Double-snare extraction technique. Two overlapping jumbo polypectomy snares (arrows) were used to grasp the stone at different angles, providing adequate leverage for extraction into the stomach.

**Figure 5:**
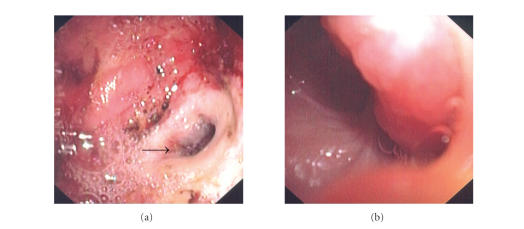
Visualization of the choledochoduodenal fistula. (a) After stone extraction, the large orifice of the fistula (arrow) can be seen in the duodenal bulb, whose mucosa is diffusely ulcerated. (b) The gastroscope passed easily through the fistula into the lumen of the gallbladder.

Three days later endoscopy was repeated, and an intracorporeal electrohydraulic lithotripter (IEHL; Northgate Technologies, Ill, USA), which was previously unavailable, was employed, as working with the Holmium: YAG laser had been only partially successful. Using a 1.9F fiber (power of 1, increased to 40; frequency of 10, increased to 30) under constant saline irrigation, IEHL was successful at shattering the outer “shell” of the stone and breaking it into two large pieces, leaving behind an extremely hard, smaller core (see Figure 3(b)). Ultimately, the majority of the stone was fragmented (Figure 3(c)) though the larger piece still could not be removed easily from the duodenal bulb. Using a “double-snare” technique, two jumbo polypectomy snares (3×6 cm, Cook Endoscopy, Ind, USA) were used to grasp the still-impacted large stone fragment at different angles and pull it into the stomach (see Figure 4). Examination of the remainder of the duodenum showed no other stones. The large cholecystoduodenal fistula was visualized, and the gastroscope easily passed into the lumen of the gallbladder (see Figure 5). Due to the length of the procedure, we chose to complete the endoscopy another day.

**Figure 6:**
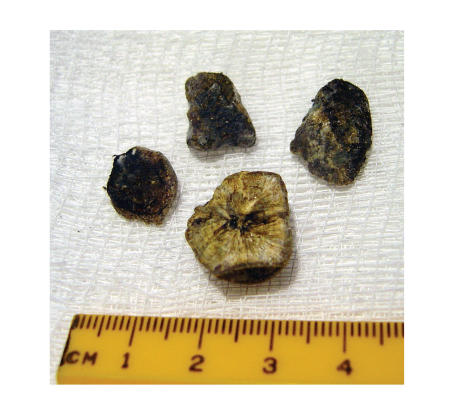
The largest stone fragments were removed perorally. The inner composition of the largest piece can be seen, measuring greater than 1 cm in diameter.

On repeat endoscopy, the largest stone fragment had again become impacted in the duodenal bulb, but was extracted by placing a biliary balloon behind it and a polypectomy snare around its center. In the stomach, the largest stones could not be crushed despite use of a mechanical lithotripter. To further break up the stones, we used the Holmium: YAG laser (1000-micron fiber for 128 joules for 427 pulses per second for a total delivery time of 2 minutes and 49 seconds) to bore multiple holes into the center of each fragment, which was then crushed with a biliary stone basket. The larger fragments were removed perorally (see Figure 6) with a Roth net (US Endoscopy, Ohio, USA), while the very small pieces were left behind to pass spontaneously. Two weeks later, a fourth endoscopy was performed to place a gastrostomy feeding tube, and no residual stones were seen in the stomach or duodenum. Additionally, the orifice of the cholecystoduodenal fistula was significantly smaller. Two months later, the patient remained asymptomatic.

## 3. DISCUSSION

Bouveret's syndrome was first described in 1896 by Leon Bouveret, a French internist and masterful diagnostician, who reported two patients with large gallstones causing gastric outlet obstruction, both of whom died [2]. This is a very rare condition, representing fewer than 5% of cases of gallstone ileus, which itself complicates cholelithiasis in only 0.3–4% of cases [1]. The condition has been associated with significant mortality despite modern surgical techniques, and is estimated to be 12–30% [3]. The first case of successful endoscopic management of Bouveret's was in 1985 [4]. Since then there have been only a few other reports of endoscopic successes, some requiring up to eight sessions [5–9] despite the use of multiple devices including mechanical lithotriptors, electrohydraulic lithotripsy, and a variety of laser lithotripters (e.g., Holmium: YAG, Rhoadmine 6G, FREDDY). Even extracorporeal shock wave lithotripsy (ESWL), another option for nonsurgical treatment, is rarely successful [10] and not always readily available. In a recent review of the literature, Lowe et al. demonstrated that more than 90%of patients ultimately require surgical management [1].

Our case is representative of the typical patient with Bouveret's, as well as the difficult challenges involved in approaching the endoscopic management. Due to multiple comorbidities and a severely ulcerated duodenum, the surgeons were hesitant to operate. The large size of the stone (more than 3 cm in diameter), its location, and the propensity for instruments to follow the fistulous tract rather than the duodenal lumen made maneuvering extremely difficult, and precluded the use of a mechanical lithotripter.

We chose the Holmium: YAG laser and IEHL because of their prior successes in treating difficult common bile duct stones, as well as their availability at our institution. We found the Holmium: YAG laser to be useful in producing initial cracks in the stone's surface; IEHL, however, seemed to be more effective in uniformly shattering the outer surface though not very effective at attacking its harder, inner core. Using both lithotripters required a significant amount of time, in part because of the minimal working space as well as the extreme care involved in avoiding further damage to the already ulcerated bulbar mucosa. Using laser or IEHL fibers of larger diameter may have improved the efficiency of lithotripsy, but these were not available to us. When approaching the large stone fragments in the stomach, we found “drilling” multiple holes with the Holmium: YAG laser to weaken the internal structure of the stone's core to be a very useful technique which then allowed us to easily crush these large fragments with a basket. We also found that to successfully extract the stone from the bulb after lithotripsy, a single instrument did not provide enough leverage or balance. Using two instruments with a double-channel gastroscope, however, was proved to be successful twice (once using two snares, once using a snare and a biliary balloon).

Another important observation in our case was that between the second and third endoscopies, one of the stone fragments left in the stomach again became impacted in the duodenal bulb. Fortunately, this happened to be quite a large fragment, for a smaller piece might have traversed the duodenum and lodged in the ileum causing a distal gallstone ileus requiring surgery. This complication has indeed been reported several times in the literature [11, 12]. We therefore recommend that caution be used when leaving stones in the stomach between treatment sessions. A nasogastric tube was used to decrease the risk of vomiting and aspirating stone fragments. Finally, two weeks after stone extraction, the size of the fistula was seen to be much smaller. Surgical closure of the fistula is not currently recommended; in fact in patients with Bouveret's undergoing enterolithotomy, the fistulas are left undisturbed and usually do not cause complications [1].

We believe our case to be the first full-length report of the successful treatment in the United States of Bouveret's syndrome with endoscopy alone, as well as a unique description of the complementary use of different lithotriptors and instruments. Our experience suggests that endoscopists faced with this clinical problem should use a double channel gastroscope, initiate stone fragmentation with IEHL as first line management, crush stone fragments that are left in the stomach to prevent ileal obstruction, and expect multiple endoscopic sessions. We anticipate that due to the aging population in the US and the epidemic of obesity, Bouveret's will likely become more common than previously reported. It is, therefore, important for endoscopists to be familiar with the multiple options available to effectively treat these difficult cases without surgery.
